# Partial pressure of oxygen-guided adrenal venous sampling in primary aldosteronism

**DOI:** 10.1210/clinem/dgag089

**Published:** 2026-03-06

**Authors:** Kei Omata, Yuta Tezuka, Hiromitsu Tannai, Yoshikiyo Ono, Hiroshi Ishihata, Hiroki Kamada, Sota Oguro, Yoshihide Kawasaki, Akihiro Ito, Yuto Yamazaki, Hironobu Sasano, Takashi Suzuki, Tetsuhiro Tanaka, Kei Takase, Hideki Katagiri, Fumitoshi Satoh

**Affiliations:** Department of Diabetes, Metabolism, and Endocrinology, Tohoku University Graduate School of Medicine, Sendai, Miyagi 980-8575, Japan; Department of Diabetes, Metabolism, and Endocrinology, Tohoku University Graduate School of Medicine, Sendai, Miyagi 980-8575, Japan; Department of Diagnostic Radiology, Tohoku University Graduate School of Medicine, Sendai, Miyagi 980-8575, Japan; Department of Diabetes, Metabolism, and Endocrinology, Tohoku University Graduate School of Medicine, Sendai, Miyagi 980-8575, Japan; Department of Periodontology and Endodontology, Tohoku University Graduate School of Dentistry, Sendai, Miyagi 980-8575, Japan; Department of Diagnostic Radiology, Tohoku University Graduate School of Medicine, Sendai, Miyagi 980-8575, Japan; Department of Diagnostic Radiology, Tohoku University Graduate School of Medicine, Sendai, Miyagi 980-8575, Japan; Department of Urology, Tohoku University Graduate School of Medicine, Sendai, Miyagi 980-8575, Japan; Department of Urology, Tohoku University Graduate School of Medicine, Sendai, Miyagi 980-8575, Japan; Department of Pathology, Tohoku University Graduate School of Medicine, Sendai, Miyagi 980-8575, Japan; Department of Pathology, Tohoku University Graduate School of Medicine, Sendai, Miyagi 980-8575, Japan; Department of Pathology, Tohoku University Graduate School of Medicine, Sendai, Miyagi 980-8575, Japan; Department of Nephrology and Hypertension, Tohoku University Hospital, Sendai, Miyagi 980-8575, Japan; Department of Diagnostic Radiology, Tohoku University Graduate School of Medicine, Sendai, Miyagi 980-8575, Japan; Department of Diabetes, Metabolism, and Endocrinology, Tohoku University Graduate School of Medicine, Sendai, Miyagi 980-8575, Japan; Department of Pathology, Tohoku University Graduate School of Medicine, Sendai, Miyagi 980-8575, Japan; Department of Nephrology and Hypertension, Tohoku University Hospital, Sendai, Miyagi 980-8575, Japan

**Keywords:** primary aldosteronism, adrenal venous sampling, partial pressure of oxygen

## Abstract

**Context:**

Adrenal venous sampling (AVS) plays a pivotal role in treatment optimization for primary aldosteronism (PA) to minimize cardiovascular risks. However, technical difficulties often hinder accurate cannulation to the adrenal veins (AVs).

**Objective:**

This study aimed to explore the distribution of partial pressure of oxygen (pO_2_) in the AV and neighboring veins, inspired by our awareness of the lighter red color of adrenal venous blood than the others.

**Methods:**

We enrolled 179 PA patients who underwent AVS from 2021 to 2024. During AVS, we collected residual blood samples from bilateral AV, hepatic (HV), inferior phrenic, and external iliac veins for blood gas analysis. Statistical analysis was conducted to evaluate pO_2_ distributions and its associations with clinical parameters.

**Results:**

Among 179 patients examined, 168 received oxygen supplementation during AVS and in those cases, the pO_2_ levels were significantly higher in the bilateral AVs than in the HV and inferior phrenic veins at baseline, whereas the levels of partial pressure of carbon dioxide were lower. Following cosyntropin stimulation, the pO_2_ levels in the AVs decreased but distribution patterns across the examined veins remained similar. The pO_2_ evaluation provided highly accurate identification of AVs both before and after cosyntropin stimulation.

**Conclusion:**

This is the first study to examine the pO_2_ dynamics in the human AV and non-AVs, demonstrating its potential to improve AVS cannulation success rates. Our findings also presented the oxygen consumption in the adrenal glands for steroidogenesis. The pO_2_ measurement is a faster, easier and less-expensive tool enhancing AVS techniques.

Primary aldosteronism (PA) is a leading cause of endocrine hypertension, accounting for 3% to 12% of all patients with hypertension in primary care settings (1). A pile of evidence elucidated that PA frequently triggered cardiovascular disease (CVD) both in blood pressure (BP)- and mineralocorticoid-dependent manners: The enhanced risks of CVD in PA were reported as 1.67 to 1.77, 2.05, 2.03 to 2.58, and 2.68 times higher for coronary artery disease, heart failure, stroke, and proteinuria, respectively, compared to those with essential hypertension (EH) (2-4). In addition, PA could lead to a higher risk of mortality than EH (5). PA-specific treatment, therefore, plays a pivotal role in mitigating the increased CVD risks as well as achieving better BP control (6). PA has been known to consist of two major subtypes: unilateral PA, mostly caused by aldosterone-producing adenomas, and bilateral PA (7). Among unilateral PA cases, a meta-analysis revealed that adrenalectomy resulted in complete clinical and biochemical outcomes in 39% and 99% of the cases, respectively, based on the Primary Aldosteronism Surgical Outcome (PASO) criteria (8). In addition, previous studies demonstrated that adrenalectomy resulted in more favorable clinical and biochemical outcomes than medical treatment with mineralocorticoid receptor antagonists (MRAs) (9, 10). However, in bilateral PA cases, administration of MRAs is a major mode of therapy to reverse the excess risks of developing comorbidities, while the efficacy of medical treatment depends on MRA titration by physicians. Therefore, whether treatment is optimized for each PA subtype or not greatly affects healthy life expectancy in patients.

Toward classification of PA patients, adrenal venous sampling (AVS) has been employed as a gold-standard procedure to differentiate unilateral PA, potentially surgery-curable form, from bilateral PA (6). The techniques of AVS have been well developed, but are still challenging due to the difficulties in cannulating AVs. In addition, the reported success rate of bilateral AV cannulation has been reported to vary widely, ranging from 60% to 99% among different institutions (11-15). A major cause of AVS failure is the difficulty in intraprocedural confirmation of AV cannulation. A couple of studies reported anatomical variation of right and left AVs (RAV and LAV) (16, 17). The RAV normally flows into the inferior vena cava (IVC), whereas we reported that 20% of PA patients who underwent AVS had a common trunk of RAV and the accessory hepatic vein (HV) (16). Those cases demonstrated various locations of the RAV orifice and angles between RAV and HV or IVC. The close proximity of the openings of the RAV and HV could therefore prevent successful RAV cannulation. Furthermore, on venograms, both veins could exhibit similar morphologies, leading to misidentification and sampling from the HV instead of the RAV. This is considered one of the causes of AVS failure. On the other hand, the LAV has been reported to form a common trunk with the inferior phrenic vein (IPV), draining blood from the diaphragm and stomach, before the LAV flows into the left renal vein in more than 90% of the relevant cases (17). Those anatomical features and individual differences sometimes resulted in misidentification of AVs even in experienced centers. Today, the selectivity index (SI) using cortisol levels in the target and reference veins is globally used to judge whether adrenal veins are appropriately cannulated (18). To improve the success rate of AVS, intraprocedural measurement of cortisol has been suggested in addition to preoperative imaging focusing on the AVs (19, 20).

Recently, we have noticed macroscopic color difference between blood specimens from the external iliac vein (EIV) and the AV in AVS. The red color of AV blood appeared to be lighter than those of the neighboring veins, including the HV and EIV. Subsequent evaluation revealed that partial pressure of oxygen (pO_2_) was higher in AV samples than in other venous samples, including HV and IVC. We, therefore, conducted the present study to prospectively investigate the pO_2_ differences among blood obtained from AVs and nearby veins, with a hypothesis that the pO_2_ measurement is an indicator for successful cannulation of AVs during the AVS procedure.

## Materials and methods

### Ethical considerations

This study protocol was developed in accordance with the tenets of the Declaration of Helsinki and approved by the Tohoku University Hospital institutional review board (2023-1-817). We obtained written informed consent from all participants for the procedures for this study.

### Eligibility of the participants for this study

To evaluate pO_2_ differences among the AV and relevant veins, we included PA patients who underwent AVS for subtyping in our center from April 2021 to March 2024. Diagnosis of PA was performed as per the PA guidelines (21, 22). Briefly, hypertensive patients underwent evaluation of plasma renin and aldosterone after sufficient withdrawal of antihypertensive agents, which interfere in the renin-angiotensin-system. Blood samples for renin and aldosterone evaluation were obtained after 30 minutes’ rest in the supine position in the morning. Screening criteria for PA were defined as a plasma aldosterone concentration (PAC) higher than 6 ng/dL along with renin suppression (plasma renin activity [PRA] < 1 ng/mL/h or its concentration [PRC] < 4 pg/mL) (23). PAC and PRC were both measured by the recently developed chemiluminescent enzyme immunoassay (23) and PRA by a commercially available enzyme immunoassay. The diagnosis of PA was established based on the results of a captopril challenge test (aldosterone-to-renin ratio > 8.2 ng/dL per ng/mL/h after 50-mg captopril loading) and/or saline infusion test (PAC > 1.2 ng/dL after 2-L saline injection) (21, 24). Those who had overt Cushing syndrome or other uncontrolled thyroid dysfunction were excluded from this study (25). BP was measured with the HEM 7120 (Omron Healthcare Co Ltd) after 5 minutes of rest. Basic clinical information of the participants was collected by reviewing their medical records.

### Adrenal imaging by computed tomography

Noncontrast and 4-phase dynamic contrast-enhanced computed tomography (CT) was primarily employed to evaluate adrenal nodules, AVs, and their neighboring veins using a 160-row multidetector CT system (Aquilion Precision; Canon Medical Systems) (16). The tube voltage was set to 120 kVp and the tube current to 310 mA. The scanner operated with a rotation time of 0.5 seconds and a pitch of 0.8. Transverse reconstructed images were acquired with a slice thickness and slice interval of 1 mm, a matrix size of 512 × 512 pixels, and a field of view of approximately 320 mm for all phases. Specifically, for the late arterial phase images, higher resolution was achieved with a slice thickness and interval of 0.25 mm, a matrix size of 512 × 512 pixels, and a field of view of approximately 240 mm. A nonionic contrast agent, containing 300 to 370 mg iodine/mL, was injected at a dose of 600 mg I per patient body weight into a peripheral vein over 25 seconds. When the attenuation value reached a preset threshold (CT value on plain CT plus 50 Hounsfield units) at the level of the abdominal aorta, early arterial-phase scanning automatically commenced (16). Late arterial-phase scanning began 13 seconds after the completion of the first scan. Venous-phase and delayed-phase scanning started 70 seconds and 3 minutes, respectively, after the initiation of contrast injection.

### Subtyping using segmental adrenal venous sampling

The procedure was performed by interventional radiologists, with endocrinologists in attendance. A 5-F or 7-F sheath was inserted into the bilateral femoral veins under ultrasonography guidance. A 5-F or 6.5-F diagnostic catheter for the LAV or RAV (Adselect, Hanaco Medical; MK adrenal type; Hanaco Medical) and a 2- to 2.9-F split-tip microcatheter (Goldcrest Neo microcatheter type OM, Medico's Hirata) were also used (26-28). First, the LAV was cannulated with the catheter. Subsequently, the HV and RAV were cannulated with the right adrenal catheter. Blood sampling was carefully conducted to obtain a minimum of 1.5 mL from the RAV, HV, and LAV (from sites before and after IPV confluence, referred to as LAV_CV_ and LAV_IPV_, respectively.) and from the right EIV using a microcatheter as needed. HV sampling was primarily performed from the middle or right HV, and occasionally from an accessory HV. Venous samples were also collected from the IPV when adrenal blood flow to the IPV was suspected. Bilateral AVS was completed within a short time frame. Subsequently, a 200-μg intravenous bolus of cosyntropin (synthetic adrenocorticotropin, ACTH) was administered, followed by a continuous infusion of 50 μg/h via a peripheral vein 30 minutes later (14, 26-28). Fifteen minutes after the initiation of ACTH stimulation, sampling from the same sites and bilateral adrenal tributary veins was conducted. Heparinization was performed to prevent coagulation inside the catheter. Basically, oxygen was administrated at 2 L/min via nasal canula throughout the AVS procedure to keep oxygen saturation over 98%.

The laterality of PA was determined based on the lateralized index under ACTH stimulation as previously reported (14). Briefly, unilateral PA was identified in the case in which the lateralized index, a quotient of the aldosterone/cortisol ratio obtained from the adrenal central vein of the dominant side divided by that of the other side, was higher than 2.6. Some cases were also categorized as unilateral PA by segmental AVS when the aldosterone/cortisol ratios were similar in bilateral adrenal central veins but significantly elevated only in a specific segment, often corresponding to a CT-detectable tumor. Successful cannulation to the AVs were confirmed based on the SI (the ratio of cortisol level in the target vein divided by that of the EIV > 5) as well as angiography during AVS.

### Measurement of steroids and partial pressure of oxygen and carbon dioxide in adrenal venous samples

As routine diagnostic procedures of AVS, PAC and serum cortisol were measured employing commercially available assays, the chemiluminescent enzyme immunoassay (Lumipulse Presto Aldosterone; Fujirebio Inc) and the electrochemiluminescent immunoassay (Elecsys Cortisol-II; Roche Diagnostics), respectively. Assessment of both hormones was performed using blood samples from the AVs and EIV, while only cortisol levels were measured in HV and IPV samples. For this study, we also performed blood gas analysis in residual venous samples collected from both AVs, HV, IPV, and EIV during AVS. The pO_2_ and partial pressure of carbon dioxide (pCO_2_) were measured employing an ABL800 FLEX (Radiometer Medical A/S). Blood gas analysis was performed with 0.2 mL of residual samples immediately after collection of the blood samples during AVS. Gas analysis within 3 minutes confirmed that pO_2_ stability remained within a 2% variation (Supplementary Fig. S1) (29).

### Statistical analysis

We employed SPSS (version 28.0: IBM Corp) for all statistical analyses performed in this study. For clinical information, parametric and nonparametric variables were shown as mean ± SD and median (an interquartile range), respectively. The comparison of blood pO_2_ or pCO_2_ levels between two veins or before and after ACTH stimulation was performed with the Wilcoxon signed rank test. In cases with missing values, we excluded the cases with missing data only for the specific statistical analysis affected. The differences of pO_2_ or pCO_2_ between a target vein and EIV were shown as ΔpO_2_ or pCO_2_, respectively, by subtracting their levels at EIV from those at a target vein. In addition, we employed the Spearman correlation to reveal the associations between venous gas status and steroid production. Subsequently, the influence of clinical parameters on pO_2_ levels was assessed with stepwise regression. Finally, receiver operating characteristic (ROC) curves were constructed to investigate the discriminative ability of pO_2_ and pCO_2_ levels in identifying AVs, based on the cases in which target parameters were available both for the AVs and their counterpart veins. The optimal cutoff value for ROC analysis was determined based on the Youden index. Statistical significance was set at *P* less than .05 for all statistical analyses performed in this study.

## Results

We enrolled 179 PA patients whose baseline characteristics are summarized in Supplementary Table S1 (29). Overall, 89 (49.7%) were men and the mean age and body mass index (BMI) were age 53.0 years and 24.9, respectively. Adrenal imaging found unilateral and bilateral adrenal tumors in 100 (55.9%) and 17 (9.5%) patients, respectively. A combination of adrenal imaging and AVS confirmed the confluences of the HV and RAV, and IPV and LAV in 30 (16.8%) and 176 (98.3%) of the patients, respectively. Based on segmental AVS, 104 (58.1%) patients were diagnosed with unilateral PA. Of these, 81 (77.9%) showed concordant laterality of hyperaldosteronism between adrenal imaging and segmental AVS, while others had bilateral adrenal tumors or no tumor on the adrenals. Of the 179 PA patients, 11 (6.1%) did not receive oxygen support during AVS. The subsequent analysis focusing on pO_2_ status was, therefore, limited to 168 patients who underwent AVS with oxygen supplementation to ensure accurate evaluation.

### Blood gas evaluation of venous samples obtained during adrenal venous sampling

#### Venous partial pressure of oxygen levels before cosyntropin stimulation

**Figure 1:**
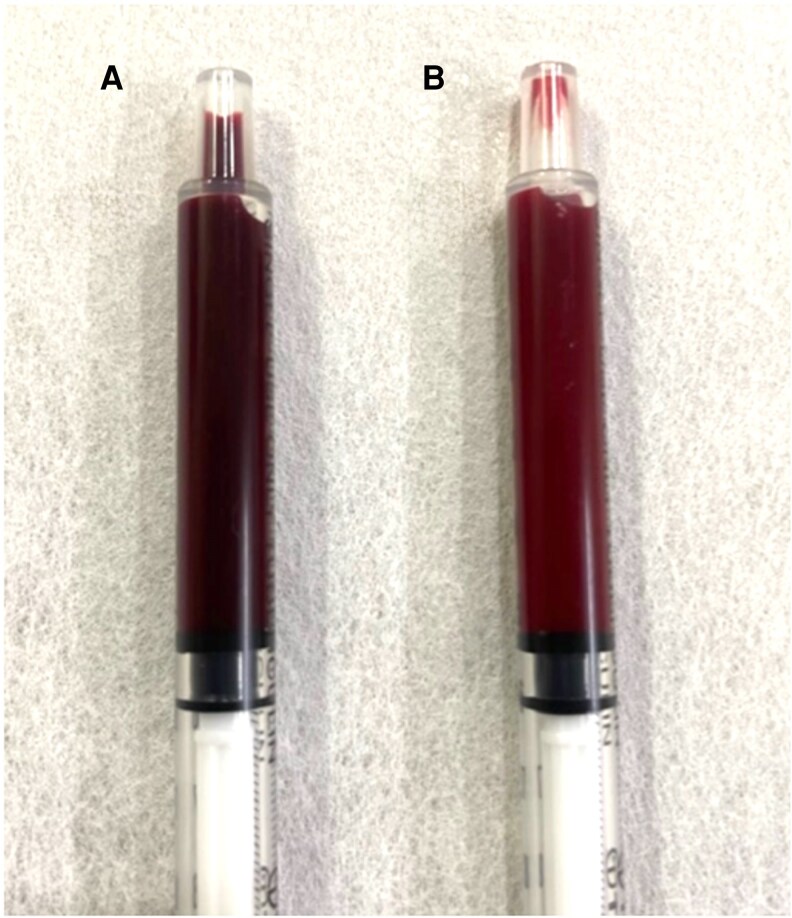
Different blood colors in the external iliac vein and the adrenal vein. Different blood colors of the A, external iliac vein and B, the adrenal vein obtained during adrenal venous sampling. Adrenal blood showed brighter red than the others.

**Figure 2:**
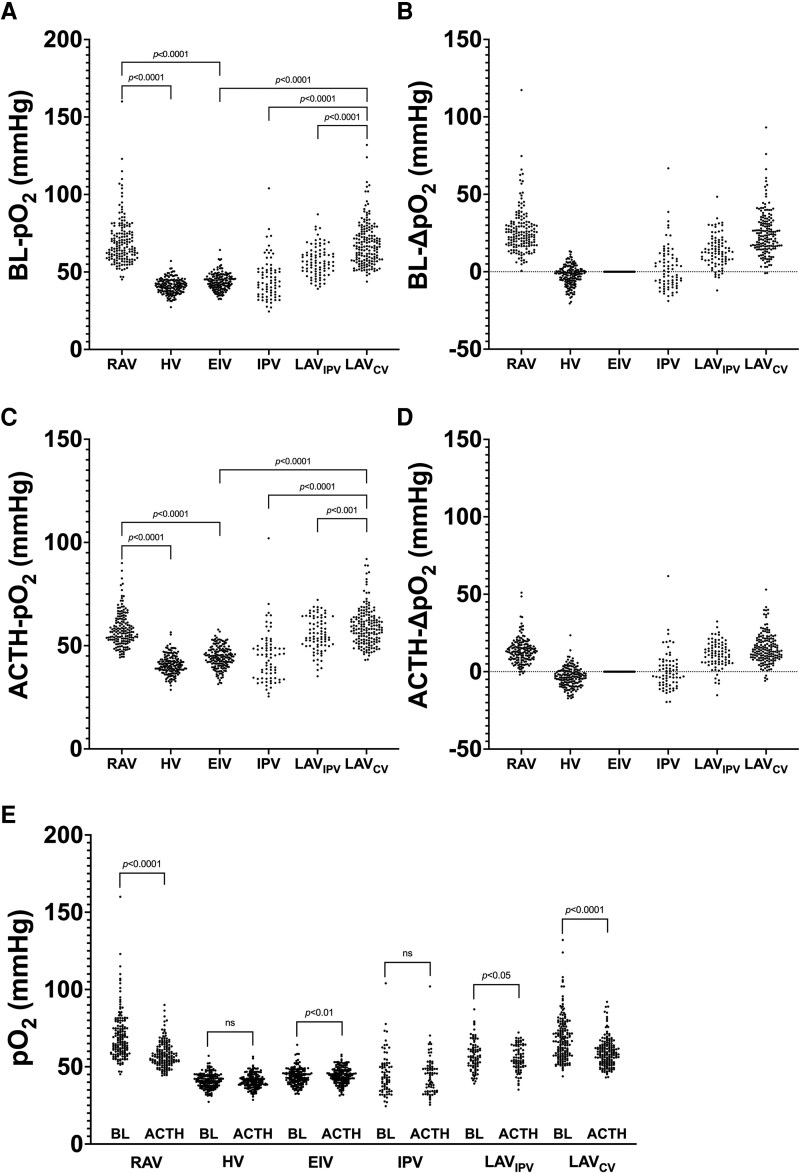
The pO_2_ evaluation in the adrenal veins and their surrounding veins. ACTH, cosyntropin stimulation; BL, baseline; EIV, external iliac vein; HV, hepatic vein; IPV, inferior phrenic vein; LAV_CV_, the central vein of left adrenal; LAV_IPV_, left adrenal vein after the confluence of IPV; pO_2_, partial pressure of oxygen; RAV, right adrenal vein. ΔpO_2_ was calculated by subtracting pO_2_ levels at EIV from those at a target vein. Baseline pO_2_ levels and calculated ΔpO_2_ in the examined veins are shown in A and B. The pO_2_ levels under cosyntropin stimulation and ΔpO_2_ are depicted in C and D. The pO_2_ levels are compared between baseline and under cosyntropin stimulation in E. ns, not significant.

A representative image illustrating the color difference between blood specimens from the EIV and the AV is shown in Fig. 1. First, we measured blood pO_2_ in the AV and neighboring veins without ACTH stimulation (Fig. 2A and Supplementary Table S2) (29). Among 168 PA patients, baseline blood samples from the RAV, HV, EIV, IPV, LAV_IPV_, and LAV_CV_ were available for gas analysis in 158 (94.0%), 165 (98.2%), 168 (100%), 73 (43.4%), 86 (51.2%), and 166 (98.8%) cases, respectively. Consistent with the lighter red blood from the AVs, both blood pO_2_ levels were significantly higher in RAV and LAV_CV_ than in EIV (66.3 [59.3-76.1] and 66.5 [57.5-76.2] mm Hg vs 43.2 [40.0-47.0] mm Hg, respectively; *P* < .0001 for both). There was no statistically significant difference in blood pO_2_ between the RAV and LAV_CV_ (*P* = .07). Among the limited cases with available gas analysis in the adrenal tributary vein, there was no significant difference in pO_2_ levels between the adrenal central and tributary veins (Supplementary Table S3) (29). Similarly, pO_2_ levels in the RAV and LAV_CV_ were significantly higher than those in their neighboring veins, HV, and IPV, respectively (40.8 [37.6-44.3] and 43.1 [35.5-51.7] mm Hg in the HV and IPV, respectively; *P* < .0001 for both) (see Supplementary Table S2) (29). Moreover, pO_2_ levels were lower in the HV than in EIV (*P* < .0001), while there was no significant difference in pO_2_ between the IPV and EIV. Thus, blood pO_2_ levels in the LAV decreased after IPV confluence (66.5 [57.5-76.2] mm Hg in the LAV_CV_ vs 56.0 [50.3-62.5] mm Hg in the LAV_IPV_; *P* < .0001). The differences in pO_2_ levels between the target veins and EIV, ΔpO_2_, are summarized in Fig. 2B. The ΔpO_2_ clearly demonstrated that blood pO_2_ levels in RAV were higher than those in the EIV in all available cases (see Fig. 2B). In addition, 164 out of 166 (98.8%) cases showed similar results between LAV_CV_ and EIV pO_2_ levels.

#### Changes in venous partial pressure of oxygen levels after cosyntropin stimulation

Next, we evaluated blood pO_2_ levels in the same veins under ACTH stimulation in 167 available cases (Fig. 2C and 2D, and Supplementary Table S2) (29). Blood pO_2_ levels significantly decreased in the RAV, from 66.3 (59.3-76.1) mm Hg to 56.6 (52.7-62.8) mm Hg (*P* < .0001), and the LAV_CV_, from 66.5 (57.5-76.2) mm Hg to 58.2 (52.7-63.8) mm Hg (*P* < .0001) after ACTH injection (Fig. 2E). In contrast, the pO_2_ levels did not change in either the HV, from 40.8 (37.6-44.3) mm Hg to 40.4 (37.8-43.4) mm Hg (not significant), or the IPV, from 43.1 (35.5-51.7) mm Hg to 45.1 (34.3-50.0) mm Hg (not significant; see Fig. 2E). In the EIV, blood pO_2_ levels slightly increased from 43.2 (40.0-47.0) mm Hg to 44.4 (41.2-47.8) mm Hg (*P* = .007). However, the dominance of blood pO_2_ levels in the AVs over those in examined non-AVs was maintained even under ACTH stimulation (see Fig. 2D).

Those trends of pO_2_ distribution at baseline and after ACTH stimulation were similar even in 11 PA cases in which O_2_ was not administered during AVS (Supplementary Fig. S2) (29). However, there were statistically significant differences in pO_2_ levels between cases with and without oxygen administration, particularly in those of the AVs at baseline (Supplementary Table S4) (29).

#### Evaluation of venous partial pressure of carbon dioxide levels before and after cosyntropin stimulation

**Figure 3:**
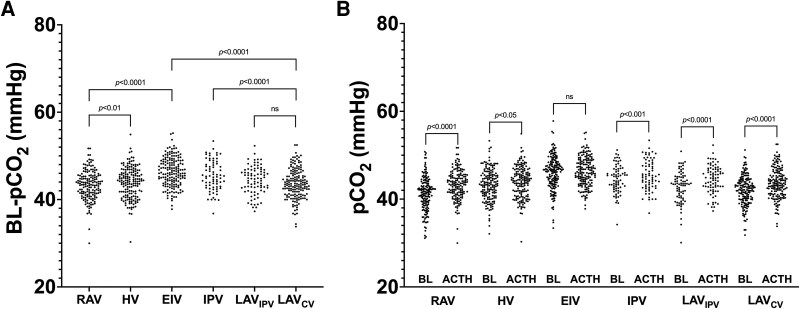
The pCO_2_ evaluation in the adrenal veins and their surrounding veins. ACTH, cosyntropin stimulation; BL, baseline; EIV, external iliac vein; HV, hepatic vein; IPV, inferior phrenic vein; pCO_2_, partial pressure of carbon dioxide; RAV, right adrenal vein; LAV_CV_, central vein of left adrenal; LAV_IPV_, left adrenal vein after the confluence of IPV. Baseline pCO_2_ levels in each vein are shown in A. Changes of pCO_2_ levels after cosyntropin injection are summarized in B. #*P* less than .05; **P less than .01; ***P less than .001; ****P less than .0001; ns, not significant.

Notably, blood pCO_2_ levels were significantly lower in the AVs compared to their neighboring veins (Fig. 3A and 3B). At baseline, the pCO_2_ levels in the RAV were lower than those in the EIV and HV, measuring 41.6 (39.0-43.7) mm Hg in the RAV vs 46.5 (43.7-48.4) mm Hg in the EIV and 43.4 (40.8-45.7) mm Hg in the HV (*P* < .0001 for both). Similarly, pCO_2_ levels were lower in the LAV_CV_ than in the EIV and IPV, with values of 42.2 (39.9-44.3) mm Hg in the LAV_CV_ compared to 46.5 (43.7-48.4) mm Hg in the EIV and 44.7 (42.6-46.9) mm Hg in the IPV (*P* < .0001 for both). Following ACTH stimulation, blood pCO_2_ levels significantly increased to 43.4 (41.3-45.5) and 43.5 (41.2-46.0) mm Hg in the RAV and LAV_CV_, respectively (*P* < .0001 for both). In addition, the initiation of ACTH resulted in a mild elevation of blood pCO_2_ levels in the HV, from 43.4 (40.8-45.6) mm Hg to 44.2 (41.1-46.3) mm Hg (*P* = .02), and IPV, from 44.7 (42.5-46.9) mm Hg to 45.2 (42.9-48.2) mm Hg (*P* = .001), but not the EIV (Fig. 3B).

#### Influential factors on venous gas status in adrenal veins

**Figure 4:**
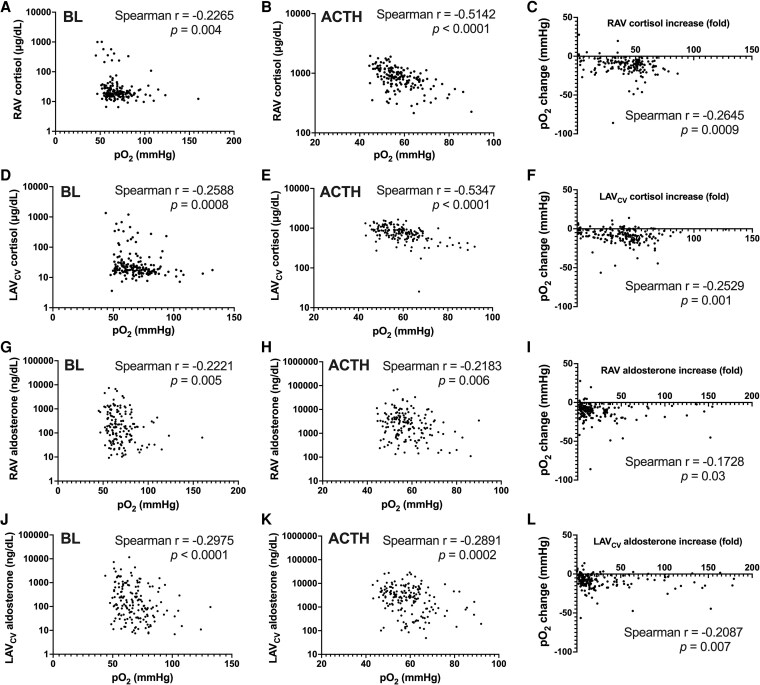
Correlations between pO_2_ and steroid production in the adrenal veins. ACTH, cosyntropin stimulation; BL, baseline; LAV_CV_, central vein of left adrenal; pO_2_, partial pressure of oxygen; RAV, right adrenal vein The pO_2_ change was calculated by subtracting pO_2_ levels under cosyntropin stimulation from their baseline levels. Cortisol or aldosterone increase was represented by dividing cortisol or aldosterone levels following cosyntropin injection by their baseline levels. The correlations between pO_2_ and cortisol production in RAV and LAV_CV_ are shown in panels A to C, and D to F, respectively. A and D show the baseline evaluation, while B and E illustrate the parameters under cosyntropin stimulation. Changes in pO_2_ and cortisol production after cosyntropin injection are displayed in C and F. For aldosterone, panels G to I and J to L represent the associations with pO_2_ for RAV and LAV_CV_, respectively. The associations between pO_2_ and aldosterone at baseline and following cosyntropin injection are shown in G and J, and H and K, respectively. Changes in pO_2_ and aldosterone production after cosyntropin injection are evaluated in I and L.

Intriguingly, in both AVs, cortisol secretion was negatively correlated with pO_2_ levels, both at baseline and under ACTH administration (Fig. 4A-4F). At baseline, the correlation between cortisol secretion and pO_2_ was weak (Spearman *r* = −0.2265 and −0.2588; *P* = .004 and .0008 in RAV and LAV_CV_, respectively) (Fig. 4A and 4D), but became moderate after ACTH stimulation (Spearman *r* = −0.5142 and −0.5347; *P* < .0001 for both in RAV and LAV_CV_, respectively) (Fig. 4B and 4E). The cortisol increase, calculated as cortisol levels after ACTH stimulation divided by baseline levels, also showed a negative correlation with pO_2_ changes between before and after ACTH initiation (Spearman *r* = −0.2645 and −0.2529; *P* = .0009 and .001 in RAV and LAV_CV_, respectively) (Fig. 4C and 4F). Similarly, PAC was also negatively correlated with pO_2_ levels (Fig. 4G-4L). The correlations of pO_2_ with PAC were similar to those with cortisol levels at baseline (Spearman *r* = −0.2221 and −0.2975; *P* = .005 and <.0001 in RAV and LAV_CV_, respectively) (see Fig. 4G and 4J), but relatively weaker under ACTH stimulation (Spearman *r* = −0.2183 and −0.2891; *P* = .006 and .0002 in RAV and LAV_CV_, respectively) (Fig. 4H and 4K). The aldosterone increase, calculated as PAC after ACTH stimulation divided by baseline levels, was also weakly but negatively correlated with the pO_2_ changes (Spearman *r* = −0.1728 and −0.2087; *P* = .03 and .007 in RAV and LAV_CV_, respectively) (Figs. 4I and 4L). Furthermore, the LAV_CV_ showed a negative correlation between aldosterone-to-cortisol ratios and pO_2_ (Spearman *r* = −0.2037 and −0.1525; *P* = .009 and .05 at baseline and after ACTH stimulation, respectively), but not the RAV (not significant). On the other hand, the HV showed a positive correlation between cortisol levels and pO_2_ only at baseline (Spearman *r* = 0.2101; *P* = .007), and the IPV showed no correlation between them (Supplementary Fig. S3) (29). In addition, no statistically significant correlation was observed between pCO_2_ and other aforementioned parameters both in the AVs and non-AVs.

Finally, multiple regression analysis revealed that BMI and ipsilateral PAC were negatively associated with pO_2_ levels in the RAV and LAV_CV_, regardless of ACTH stimulation (Supplementary Table S5). Additionally, age and ipsilateral cortisol secretion were confirmed as influential factors on pO_2_ levels in the LAV_CV_ after ACTH stimulation and RAV for both statuses. Neither PA laterality nor the presence of an adrenal tumor was associated with pO_2_ status.

### Application of blood gas analysis on detection of adrenal veins

#### Receiver operating characteristic curve analysis of venous partial pressure of oxygen in distinguishing adrenal veins

**Figure 5:**
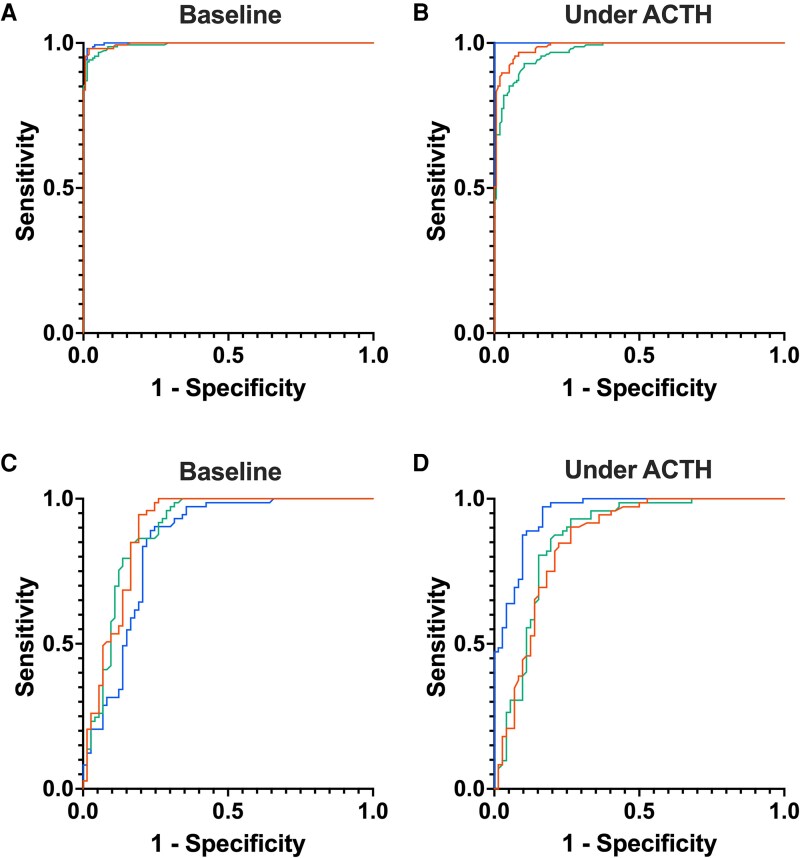
Receiver operating characteristic curve analysis for the discriminative ability of pO_2_ for the identification of adrenal veins. ACTH, cosyntropin stimulation; BL, baseline. The ability of partial pressure of oxygen (pO_2_) to distinguish right adrenal vein from hepatic vein, or left adrenal vein from inferior phrenic vein, was assessed in A and B and C and D, respectively. A (N = 154) and C (N = 73) illustrate the discriminative ability of pO_2_ at baseline, while B (N = 155) and D (N = 72) show this ability under cosyntropin stimulation, respectively. Blue, red, and green lines indicate selectivity index, actual pO_2_ and ΔpO_2_, respectively. ΔpO_2_ was calculated by subtracting pO_2_ levels at external iliac vein from those at a target vein.

Finally, we investigated the discriminative ability of venous pO_2_ and pCO_2_ in identifying AVs (Fig. 5). At baseline, ROC curves demonstrated excellent performance both of actual pO_2_ and ΔpO_2_ in differentiating the RAV from HV, comparable to the SI (Fig. 5A). Optimal cutoff values for RAV detection based on the Youden index were determined as 1.64 (sensitivity 0.981, specificity 0.987), 50.9 mm Hg (sensitivity 0.981, specificity 0.981), and 10.2 mm Hg (sensitivity 0.942, specificity 0.981) for the SI, actual pO_2_ and ΔpO_2_, respectively (Table 1). For actual pO_2_, cutoff values of 45.2 and 57.3 mm Hg showed a sensitivity of 1.000 and specificity of 1.000 for RAV detection, respectively. In addition, higher ΔpO_2_ values greater than 0.6 and 13.5 mm Hg were associated with a sensitivity of 1.000 and a specificity of 1.000 for the RAV, respectively, as well. After ACTH stimulation, a higher SI exceeding 9.4 demonstrated a sensitivity and specificity of 1.000 for RAV detection (Fig. 5B). The evaluation of actual pO_2_ and ΔpO_2_ also proved helpful for RAV cannulation even under ACTH stimulation (see Table 1). An actual pO_2_ greater than 47.2 mm Hg exhibited a sensitivity of 0.955 and specificity of 0.929, while a ΔpO_2_ greater than 3.9 mm Hg demonstrated a sensitivity of 0.929 and specificity of 0.897. However, the performance of actual pO_2_ and ΔpO_2_ was inferior to that of the SI (*P* < .01).

**Table 1:** The discriminative ability of partial pressure of oxygen for adrenal veins

For RAV detection									
	Actual pO_2_ (mm Hg)			ΔpO_2_ (mm Hg)			SI		
**At baseline**									
AUC	0.996 (0.993-1.000)			0.994 (0.988-0.999)			0.998 (0.995-1.000)		
Cutoffs	>45.2	>50.9	>57.3	>0.6	>10.2	>13.5	>1.39	>1.64	>2.16
Sensitivity	1.000	0.981	0.838	1.000	0.942	0.851	1.000	0.981	0.844
Specificity	0.838	0.981	1.000	0.708	0.981	1.000	0.929	0.987	1.000
**Under ACTH stimulation**									
AUC	0.987 (0.978-0.996)			0.971 (0.956-0.986)			1.000 (1.000-1.000)		
Cutoffs	>44.2	>47.2	>56.6	>−1.9	>3.9	>13.9	>9.4		
Sensitivity	1.000	0.955	0.497	1.000	0.929	0.452	1.000		
Specificity	0.806	0.929	1.000	0.626	0.897	1.000	1.000		

For LAV_CV_ detection									
	Actual pO_2_ (mm Hg)			ΔpO_2_ (mm Hg)			SI		
**At baseline**									
AUC	0.899 (0.844–0.953)			0.892 (0.837–0.948)			0.847 (0.781–0.914)		
Cutoffs	>51.2	>52.7	>114	>6.2	>13.9	>71.4	>1.24	>2.15	>23.8
Sensitivity	1.000	0.945	0.027	1.000	0.849	0.027	1.000	0.904	0.082
Specificity	0.740	0.808	1.000	0.658	0.836	1.000	0.342	0.753	1.000
**Under ACTH stimulation**									
AUC	0.858 (0.794–0.921)			0.868 (0.805–0.931)			0.951 (0.919–0.983)		
Cutoffs	>43.0	>49.1	>103	>−5.3	>4.3	>62.7	>14.3	>20.4	>45.5
Sensitivity	1.000	0.903	0.000	1.000	0.931	0.000	1.000	0.972	0.472
Specificity	0.472	0.736	1.000	0.319	0.736	1.000	0.694	0.833	1.000

ΔpO_2_ was calculated by subtracting pO_2_ levels at external iliac vein from those at a target vein. The ability of each parameter to distinguish the RAV from hepatic vein or LAV_CV_ from the inferior phrenic vein was investigated. AUC is presented with a 95% CI.

Abbreviations: ACTH, cosyntropin; AUC, area under the curve; LAV_CV_, central vein of left adrenal; pO_2_, partial pressure of oxygen; RAV, right adrenal vein; SI, selectivity index.

Similarly, actual pO_2_ and ΔpO_2_ were useful indicators for LAV_CV_ cannulation (Fig. 5C and 5D, and Table 1). In baseline gas analysis, optimal cutoff values of SI (>2.15), actual pO_2_ (>52.7 mm Hg) and ΔpO_2_ (>13.9 mm Hg) showed sensitivities of 0.904, 0.945, and 0.849, and specificities of 0.753, 0.808, and 0.836, respectively. Actual pO_2_ and ΔpO_2_ had larger areas under the curve (AUCs) than the SI (Fig. 5C), though the differences were not statistically significant. ACTH stimulation clearly enhanced the discriminative ability of the SI in identifying LAV_CV_, while diminishing the performance of actual pO_2_ and ΔpO_2_ (Fig. 5D). Throughout the investigation of blood gas analysis for AVS, the pCO_2_ and ΔpCO_2_ levels have less discriminative power in distinguishing the AVs from the non-AVs, despite showing significant differences in pCO_2_ between those veins (Supplementary Fig. S4) (29).

#### Representative cases illustrating the potential of partial pressure of oxygen as a guide in adrenal venous sampling

##### Case 1: discrimination of the right adrenal vein from the hepatic vein

**Figure 6:**
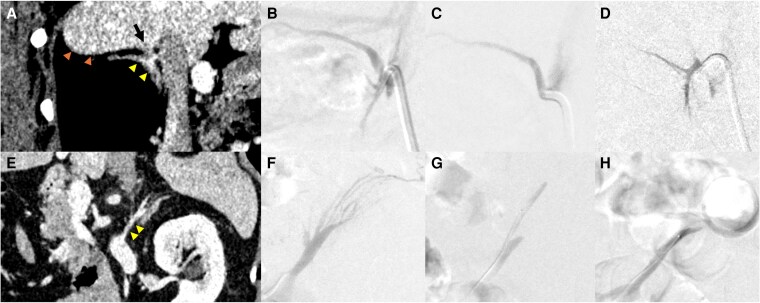
Adrenal imaging and venous angiography of representative cases. A to D and E to H show representative images of computed tomography (CT) and venous angiography from a 56-year-old and a 55-year-old man diagnosed with primary aldosteronism (cases 1 and 2), respectively. In case 1, preoperative CT revealed a confluence of the right adrenal vein and hepatic vein (A, yellow and orange arrowheads indicate the right adrenal and the liver, respectively. A black arrow marks the accessory hepatic vein, which flows into the right adrenal vein). B, Adrenal venous sampling (AVS) confirmed this confluence. The right adrenal and hepatic veins were separately selected by a catheter (C and D, respectively) and the venous partial pressure of oxygen levels at baseline were measured as 69.9 and 45.1 mm Hg, respectively. In case 2, enhanced CT visualized the left adrenal vein (E, yellow arrowheads). F, AVS identified the left adrenal vein with 3 main branches. After exclusion of the proximal one as the inferior phrenic vein, the middle and distal branches were individually cannulated for blood sampling (G and H, respectively), confirming the distal one as the left adrenal central vein based on the selectivity index. The blood partial pressure of oxygen level at baseline was measured as 37.1 mm Hg in the middle vein and 57.7 mm Hg in the distal vein.

This was the PA case of a 56-year-old man who has a common trunk of RAV and HV. Enhanced CT pointed out the possibility of a confluence of the RAV and HV prior to AVS (Fig. 6A). For confirmation of the laterality of PA, we performed AVS in which venous angiography detected a candidate for RAV shown in Fig. 6B. Blood samples for the assessment of pO_2_ as well as steroids were collected by sequentially inserting a catheter into the superior and inferior branches of the vein to verify whether those veins were draining blood from the right adrenal gland (Fig. 6C and 6D). The pO_2_ level was higher in the inferior branch (69.9 mm Hg) than in the superior branch (45.1 mm Hg) comparable to the trend of SI (Table 2). Under ACTH stimulation, the SI significantly elevated in the inferior branch (from 1.76 to 28.8), but not in the superior branch (from 1.09 to 1.34). Taken together with the findings of preoperative CT, we identified the inferior and superior branches as the RAV and HV, respectively. The pO_2_ levels maintained their dominance in the RAV over HV even after ACTH injection.

**Table 2:** Venous parameters of representative cases illustrating partial pressure of oxygen–guided adrenal venous sampling

Case 1: discrimination of RAV from HV						
	EIV (reference)		HV (shown as Fig. 6C)		RAV (shown as Fig. 6D)	
	Baseline	After ACTH	Baseline	After ACTH	Baseline	After ACTH
Cortisol, μg/dL	9.6	20.9	10.5	28	16.9	602
SI	N/A	N/A	1.09	1.34	1.76	28.8
pO_2_,mm Hg	49.1	42.1	45.1	42.1	69.9	54.1

Case 2: identification of LAV_CV_ among a few candidates								
	EIV (reference)		LAV_IPV_		Middle vein (shown as Fig. 6G)		Distal vein (shown as Fig. 6H)	
	Baseline	After ACTH	Baseline	After ACTH	Baseline	After ACTH	Baseline	After ACTH
Cortisol, μg/dL	7.6	14.1	10.8	135	8.9	32	24.5	591
SI	N/A	N/A	1.42	9.57	1.17	2.27	3.22	41.9
pO_2_, mm Hg	40.2	43	44.7	47.3	37.1	35.7	57.7	53.6

Abbreviations: ACTH, cosyntropin; EIV, external iliac vein; HV, hepatic vein; IPV, inferior phrenic vein; LAV_CV_, central vein of left adrenal; LAV_IPV_, left adrenal vein after the confluence of inferior phrenic vein; N/A, not assessed; pO_2_, partial pressure of oxygen; RAV, right adrenal vein; SI, selectivity index.

##### Case 2: identification of left adrenal vein among several candidate veins

The second case was a 55-year-old PA patient whose adrenal imaging detected a few candidates for LAV_CV_. Preoperative CT with contrast media clearly indicated the LAV, with 3 main branches suspected to drain blood from the left adrenal gland (Fig. 6E). We considered the proximal one coursing upward as IPV but could not identify which of the 2 other veins was the LAV_CV_ preoperatively. During AVS, we obtained blood samples from the 2 veins for the measurement of cortisol and pO_2_ (Fig. 6F). The SI and pO_2_ levels are summarized in Table 2. In the middle branch (Fig. 6G), the SI and pO_2_ level were 1.17 and 37.1 mm Hg, respectively, while those parameters were higher in the distal branch (3.22 and 57.7 mm Hg, respectively; Fig. 6H). After ACTH initiation, the SI increased to 2.27 and 41.9 in the middle and distal ones, respectively, resulting in confirmation of the distal vein as the LAV_CV_.

## Discussion

In our present study, we first demonstrated the difference in pO_2_ status among the AVs and their surrounding veins, which could also be clinically applicable for identification of AVs in AVS. Of note, pO_2_ status in the AVs was significantly negatively associated with steroid production from the adrenal tissue and BMI. Venous gas analysis in both before and after ACTH stimulation indicated adrenal oxygen consumption for steroid hormone synthesis.

Oxygen is one of the most critical elements for cellular activities. A sufficient supply of oxygen enables living cells to produce adenosine triphosphate, the primary energy source of the cells, and water through cellular respiration occurring in the mitochondria (30). In the adrenal gland, this aerobic reaction also supports ACTH-dependent steroidogenesis by enhancing the expression of steroidogenic enzymes using adenosine triphosphate (31). Results of our present study also demonstrated that pO_2_ levels in the AVs were significantly higher than those in the other veins examined. These findings all suggested that adrenal consumption of oxygen was relatively small, compared to the other organs such as the liver, stomach, and lower limb. However, the differences in oxygen consumption between the adrenal glands and others have not been studied in humans or animals. One exception was an animal study in which adrenal oxygen consumption was slightly lower than that of the liver in rabbits (2.53 ± 0.25 vs 3.05 ± 0.77 μL/h/mg dry weight) (32). In addition, the high vascularity of the adrenal glands (33) could also contribute to the higher pO_2_ levels observed in the AVs. In humans, arterial blood supply to bilateral adrenal glands typically originated from the abdominal aorta, renal, and phrenic arteries (34). The adrenal glands were reported to receive up to 0.5% of the total cardiac output according to the results of animal studies (35-37), while the adrenal glands are also one of the organs receiving the highest blood flow per unit of weight (36). Therefore, the adrenal glands are considered to constantly receive an abundant supply of oxygen.

In the adrenal cortex, oxygen is also involved in the steroidogenic process, particularly in oxidation reactions. Several steroidogenic enzymes, including aldosterone synthase, use oxygen within the mitochondria and the smooth endoplasmic reticulum during the transformation of cholesterol into steroid hormones (38). In addition, chromaffin cells in the adrenal medulla also require oxygen for catecholamine synthesis during the hydroxylation step (39). During hormone synthesis, these enzymes also generates reactive oxygen species, which are scavenged by antioxidant enzymes such as copper and zinc and manganese superoxide dismutase (SOD) (40). In those SODs, copper and zinc-SOD acted as a scavenger of toxic superoxide radicals generated during steroidogenesis, and manganese-SOD during catecholamine production (40).

Regarding adrenal oxygen consumption, a couple of animal studies reported that slices of dog and pig adrenal tissues exhibited baseline oxygen uptake rates of 84.4 and 86 mm^3^/100 mg weight/h at baseline, respectively (41, 42). Both studies also demonstrated an increased oxygen uptake by 20 to 40 mm^3^/100 mg weight/h following administration of ACTH (41, 42). In addition, oxygen availability has been identified as a regulatory factor in steroidogenesis (43-46). An in vitro study using bovine adrenocortical cells demonstrated that low pO_2_ conditions (80-90 mm Hg) significantly decreased aldosterone production at baseline (−31%) and in response to angiotensin II (−45 to −55%), cyclic adenosine monophosphate (−48 to −54%), and ACTH (−13 to −33%), compared with normal pO_2_ conditions (140-150 mm Hg) (43). Similarly, oxygen-dependent regulation of steroidogenesis has also been observed in human tissue experiments, particularly under stimulated conditions (46). In accordance with these reports, we observed a set of decreased pO_2_ and increased pCO_2_ levels in the AVs after ACTH administration. In addition, pO_2_ changes before and after ACTH stimulation were significantly negatively correlated with production both of aldosterone and cortisol in the AVs. Results of our present study are, therefore, interpreted as oxygen consumption during ACTH-dependent steroid synthesis in the adrenal glands. As ACTH also drives the production of other mineralocorticoids, glucocorticoids, and androgens in PA (47), the synthesis of these unevaluated adrenal steroids could possibly account for the relatively low correlation coefficients between changes in pO_2_ and aldosterone or cortisol levels observed in this study, but it awaits further investigations for clarification. These findings also suggest that the rich vascularization of the adrenal glands provides sufficient oxygen to support prompt steroid hormone synthesis in response to physiological demand.

Consequently, baseline pO_2_ levels in the AVs were considered higher than in other veins due to the relatively abundant oxygen supply. This characteristic was used in the present study to assess successful cannulation to the AVs during AVS. Regarding the observed negative effect of BMI on AV pO_2_ levels, several clinical studies have reported that obesity is associated with lower oxygen saturation, and lower maximal oxygen uptake during exercise (48-51). The mechanisms underlying this association have been unclear, but the reduction of expiratory residual volume under obesity is considered one of the contributing factors (52). The possible association between adrenal oxygen consumption and body size remains unknown at this juncture, and therefore further studies are warranted to expand our understanding about the mechanisms of oxygen consumption in adrenal cortical cells and influential factors.

Notably, our study first indicated that pO_2_ evaluation was a promising tool or pathfinder for identifying AVs during AVS. Nearly 60 years have passed since AVS was first proposed for the subtype determination of PA (53). However, cannulating the AVs is still clinically challenging. Today, the combination of preprocedural imaging of AVs and the development of customized catheters has led to higher AVS success rates than ever before (14, 16). Nevertheless, intraprocedural judging whether the cannulated vessel is the target AV based on angiography is sometimes difficult, particularly for operators with limited experience (19). Successful cannulation to the AVs is worldwide defined by achieving a higher SI with specific cutoff values that depend on sampling conditions and regions (14, 18, 21, 22). Recently, a new assay of cortisol with a shortened measurement time has been developed for the quick assessment of AV cannulation (19). This immunochromatographic assay measures plasma cortisol concentrations quantitatively within 6 minutes, increasing successful AVS rates from 54% to 93% judged by the SI in a multicenter study (19). With the findings from our present study, we propose another, easy-to-use approach to the judgment of AV selection using pO_2_ levels in the cannulated vein. Blood gas analyzers are widely available in hospitals, including in the inpatient unit, catheter laboratory, and emergency department. Blood gas analysis needs only a small blood sample and can be completed within 1 minute. Results of our present study also demonstrated that pO_2_ levels were significantly higher in the AVs than in their counterpart ones, resulting in a great ability of pO_2_ assessment to identify the AVs compatible with the SI. In particular, the measurement of pO_2_ levels clearly differentiated the RAV from the HV. This advantage of pO_2_ measurement is indeed considered clinically valuable, as the success rate of RAV cannulation tends to be lower than that of the LAV (18). Thus, AVS operators can easily and repeatedly use the technique to confirm whether the catheter is properly inserted into the AVs until successful cannulation is achieved.

The present study proposes pO_2_-guided AVS and provides novel insight into adrenal oxygen consumption, but there are a few limitations to be considered for interpreting our findings. First, our AVS protocol includes oxygen administration to settle patients' breathing for the efficient performance of AVS. This medical procedure might alter the pO_2_ levels in the examined veins and the cutoff values for the AVs. Second, the cases in which we assessed the pO_2_ levels in the IPV were limited due to the low clinical necessity, which influenced the ROC curve for differentiating the LAV_CV_ from the IPV. Finally, this is a single-center study, but not a multicenter study. A future study collaborating with multiple international centers is expected to help the clear understanding of the utility of pO_2_ assessment for PA subtyping.

In conclusion, the first awareness of different blood colors between AVs and non-AVs brought us to the present study, in which we successfully demonstrated the different distributions of pO_2_ levels in the AVs and their neighboring veins. Blood gas analysis is a faster, easier, and less-expensive tool to identify the AVs during AVS. From bedside to bench, we also observed oxygen consumption in the adrenal glands, referred to as “adrenal breathing.” Our findings should advance both clinical practice and research in the field of adrenal endocrinology.

## Data Availability

Data presented in this study are available from the corresponding authors on request.

## Abbreviations

ACTH: cosyntropin (adrenocorticotropin)
AUC: area under the curve
AV: adrenal vein
AVS: adrenal venous sampling
BMI: body mass index
BP: blood pressure
CT: computed tomography
CVD: cardiovascular disease
EH: essential hypertension
EIV: external iliac vein
HV: hepatic vein
IPV: inferior phrenic vein
IVC: inferior vena cava
LAV: left adrenal vein
LAV_CV_: central vein of left adrenal
LAV_IPV_: left adrenal vein after the confluence of IPV
MRA: mineralocorticoid receptor antagonist
PA: primary aldosteronism
PAC: plasma aldosterone concentration
pCO_2_: partial pressure of carbon dioxide
pO_2_: partial pressure of oxygen
PRA: plasma renin activity
PRC: plasma renin concentration
RAV: right adrenal vein
ROC: receiver operating characteristic
SI: selectivity index
SOD: superoxide dismutase
